# Delivering Patient Education in Healthcare Organizations: An Integrative Review of the Nursing Administrative Actions

**DOI:** 10.1155/2024/1296011

**Published:** 2024-03-07

**Authors:** Elina Linnavuori, Saija Inkeroinen, Anna Kostylev, Mariella Seel, Milka Virmajoki, Heli Virtanen, Helena Leino-Kilpi

**Affiliations:** ^1^Department of Nursing Science, University of Turku, Turku, Finland; ^2^Department of Evidence-Based Medicine and Evaluation, University of Krems, Krems, Austria; ^3^Turku University Hospital, Turku, Finland

## Abstract

**Aim:**

To analyze the literature on nursing administrative actions related to patient education to inform healthcare organizations for the development of patient education and identify the knowledge gaps for future research.

**Background:**

Patient education is a patient's right, yet numerous organizational factors can hinder the effective delivery of patient education. Nursing administrative actions can mediate these factors, but little is known about nursing administration in patient education. *Evaluation.* A systematic integrative review was conducted in October 2022 on five databases (CINAHL (Ebsco), PubMed, Web of Science, ABI/Inform, and Business Source Complete). No time limitations were set. Empirical research articles reporting nursing administrative actions related to educational actions as a main topic were screened and evaluated. The data analysis was based on a constant comparison method. *Key Issues*. There were 3,110 studies identified, of which five quantitative and four qualitative studies were included. Three main themes were generated from the data describing nursing administrative actions related to patient education: (1) strengthen the commitment to patient education, (2) ensure the necessary resources for patient education, and (3) enhance patient education policies.

**Conclusion:**

This review provides insights into nursing administration in patient education, focusing on commitment, resources, and policies. It not only highlights the critical role of nurse administrators but also calls for further research to assess the impact of these actions. *Implications for Nursing Management.* The findings of this review will be useful for nurse administrators by providing knowledge about nursing administrative actions in patient education and underlining the need for them to pay more attention to it. The implications for nursing management also include supporting further research in the field.

## 1. Introduction

Patient education is a patient's right [1] and is essential for supporting the empowerment of people in promoting their health and managing their health problems. Patient education is both an “art” and a “science,” meaning it is nuanced and evidence-based, and a component of high-quality care [2]. Even though education is considered a core activity of nursing, executing excellent patient education may be demanding due to the need for a comprehensive and patient-centered approach, incorporating both the “art” and “science” aspects [2]. Patient education is described as a process focusing on both teaching and learning [3]. Terms related to patient education have been identified in the nursing literature despite some differences in these terms, for example, patient teaching, health education [3], health information, counseling, and health promotion [4]. On this basis, we use the term patient education in this review, but the study also considered other terms in the literature search phase, recognizing their interchangeable use with patient education.

A variety of organizational factors have been identified that should be improved in the delivery of patient education [5, 6]. For example, nurses have reported inadequate organizational support, unclear job descriptions [6], and a lack of resources and time [5]. These studies discuss the important role of the nursing manager in supporting patient education. Recently, practice standards have been developed for patient education in nurse-led clinics, and several of these statements address management's role in patient education [7]. Despite the importance of organizational factors and managerial support for patient education, little is known about nursing management in this core activity of nursing.

Administrator refers to a person who is responsible for carrying out the administration of an organization [8]. In the context of healthcare, the American Nurses Association (ANA) [9], for example, defines a nurse administrator as a person “who orchestrates and influences the work of others in a defined environment, most often healthcare-focused, to enhance the shared vision of an organization or institution” (p.3). Administrators can work at different administration levels of the organization [10]. Administrators at the first and middle levels oversee clinical units at the microlevel, while administrators at the executive level handle top-level patient care administration [11]. There is variation in the international literature on the titles of nurse administrators because of differences in healthcare systems. These titles include nurse manager, ward manager, charge nurse, head nurse, nurse director, supervisor, and nursing officer [12]. In this review, we use the term nurse administrator to cover all these different level administrators in the organizations, assuming their importance for patient education.

Nurse administrators' responsibilities include aspects of both leadership and management which often overlap, such as human resource management, collaboration, nursing development, financial management, and planning and evaluation of actions [13]. Several different leadership styles can also be identified in the work of nurse administrators [14]. In this review, we use the term nursing administration to include both leadership and management. The role of the nurse administrator is important for patient safety and quality of care [15]. It also influences missed nursing care [16]. The work of nurse administrators has an impact on the whole nursing process. Nurse administrators play a crucial role in patient education, and systematic research is required to identify the administrative actions relating to patient education.

## 2. Aim

The aim of the study was to analyze the literature on nursing administrative actions related to patient education to inform healthcare organizations about the development of patient education and identify the knowledge gaps for future research.

The research questions were as follows:

1. What are the nursing administrative actions related to patient education?
2. What is the quality of the studies on the nursing administrative actions related to patient education?

## 3. Methods

### 3.1. Design

The study design is a systematic integrative review. A guideline by Whittemore and Knafl [17] for the integrative review was followed and complemented by the Preferred Reporting Items for Systematic Reviews and Meta-Analyses (PRISMA) guidelines [18] according to the protocol [19].

### 3.2. Literature Search

The search was guided by the aim and research questions. Predefined inclusion and exclusion criteria related to the topic of interest, research design, and language were followed throughout the literature search process (see Table 1).

The search was completed on 17 October 2022 on five databases: CINAHL (Ebsco), PubMed, Web of Science, ABI/Inform, and Business Source Complete. Furthermore, the reference lists of the included reports were examined by two researchers. The search terms were formulated based on the PICo framework: the population was administrators, the interest was patient education, and the context was nursing. A wide range of related keywords were used to obtain comprehensive information on the topic. The search strategy was based on the eligibility criteria and revised for each database (Table 2). The search was limited to the English language in all databases. Where possible, only peer-reviewed reports were included (i.e., in CINAHL, ABI/Inform, and Business Source Complete). There was no time limit for the inclusion studies. Prior to the systematic search, the research team repeatedly tested and revised the search strategy in close collaboration with an information specialist at the University of Turku.

The reports were transferred, and the duplicates were removed in the reference management software Zotero, Corporation for Digital Scholarship (version v6.0.15). Two researchers screened each record independently (title, abstract, and the full text). Any disagreements between screeners were resolved in discussions with the research team.

### 3.3. Data Analysis

The data analysis was based on a constant comparison method consisting of four steps as reported by Whittemore and Knafl [17]. The analysis was performed using the Methods and the Results sections of the studies. First, in the data reduction, one researcher collected the data from the included reports in a data extraction table, which included information about the author(s), year of publication, title, location, study design, the purpose of study, sample, context, data analysis, and results. Next, each study was examined for sentences and phrases describing the administrative actions in patient education in the context of nursing from the perspectives of different data sources, such as head nurses, supervisors, and nurses. Administrative actions related to patient education delivered by physicians were not included. The actions identified were extracted verbatim from each of the studies. The collection process was guided and monitored by the research team. Subsequently, data were organized and coded into a data display matrix. Data comparison was conducted by comparing and grouping coded phrases of nursing administrative actions related to patient education based on similarities of actions. The codes were gradually clustered into subthemes and finally merged into overall main themes [17].

Data quality was assessed independently by two researchers using the Joanna Briggs Institute Critical Appraisal Tools [20, 21]. The appropriate tool was chosen based on the study design of each report (Checklist for Analytical Cross-Sectional Studies or Qualitative Research). Any disagreements in data evaluation were resolved by the research team. No report was excluded based on the data evaluation.

## 4. Results

**Figure 1:**
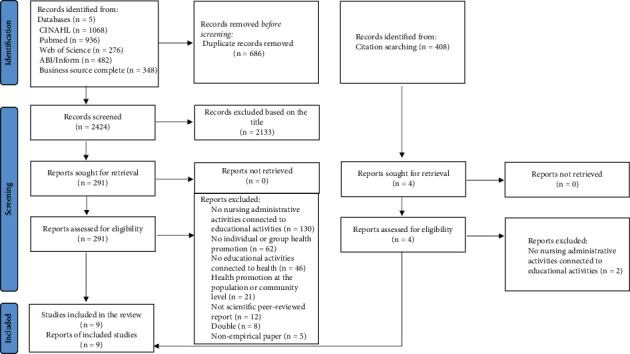
PRISMA flow diagram [18].

There were 3,110 studies identified from five databases and 408 studies from citation searches. After the removal of duplicates, 2,424 studies were screened for eligibility. The title screening process excluded 2,133 studies, 295 studies were retrieved first at the abstract level, and 55 studies were retrieved in full text. Finally, nine studies focusing directly on nursing administrative actions related to patient education met the inclusion criteria (Figure 1).

### 4.1. Characteristics of the Included Studies

The nine selected studies were published between the years 2001–2019. Five studies used a descriptive cross-sectional design [22–26], and four used qualitative designs [27–30]. The study's characteristics are presented in Table 3.

The studies were conducted in five different countries: Four in Iran [22–25, 27], two in Sweden [24, 28], one in Finland [26], one in the Netherlands [28], and one in the United States [29]. Most of the studies (*n* = 7) had a hospital environment as the study setting [22–25, 27, 28, 30]. Other study settings were adult acute psychiatric hospitals [26] and telephone advice nursing services [29], and one study was set in primary and municipal care, in addition to hospitals [24].

Nursing administrative actions related to patient education were identified from different perspectives. In most of the studies (*n* = 8), the respondents were administrators: nursing managers, head nurses or supervisors [22, 23, 25, 26, 28, 29], or patient education officers [30]. Four of these studies also included other respondents, such as nurses (*n* = 3) [25, 27, 29], documents, and websites [30]. One study reported exclusively nurses as respondents [24]. In some of these studies, the respondents also included patients [25] and physicians [25, 29]. However, the patients' and physicians' perspectives did not directly describe the relation between nursing administrative actions and patient education by nurses, and their perspectives have, therefore, not been taken into account in the final analysis of this review.

Information on nursing administrative actions related to patient education was collected through questionnaires [22–26, 29], interviews [25, 27–30], and observations [27] of respondents. In addition, websites and documents were analyzed [30].

### 4.2. Quality of the Selected Studies

Based on our quality assessment [20, 21], the quality varied among both cross-sectional and qualitative study designs (Table 3). Most commonly, there were disparities in reporting in terms of strategies to deal with confounding factors [22, 23, 25, 26] and criteria for inclusion in the sample [23, 25, 26]. All qualitative studies lacked a statement of the researcher's cultural or theoretical location [27–30]. One qualitative study clearly reported a philosophical perspective [28].

### 4.3. Nursing Administrative Actions Related to Patient Education

As a result of the analysis, three main themes describing nursing administrative actions related to patient education were identified: (1) strengthen the commitment to patient education, (2) ensure the necessary resources, and (3). enhance patient education policies. Each main theme includes several subthemes, identified from three different perspectives: nurse administrators, nurses, and policy papers. Main themes, subthemes, codes, and original phrases describing nursing administrative actions related to patient education are presented in Table 4.

The main themes (*n* = 3), subthemes (*n* = 9), and different perspectives are described below in detail. For each theme, the different perspectives are presented in order: first, the nurse administrators' perspectives, followed by the perspectives of other respondents such as nurses, or if the respondents were not specified in the study, as a common opinion.

#### 4.3.1. Theme 1: Strengthen the Commitment to Patient Education

The first theme describes nursing administrative actions to strengthen the commitment to patient education including three subthemes: enhancing motivation for patient education [22, 27, 29], prioritizing patient education [25, 27], and providing support [24, 28, 29]. These subthemes describe the strengthening of nursing, administrative, and organizational commitment to patient education in terms of reported development targets, lacking issues, and important aspects.

*(1) Enhancing Motivation for Patient Education*. Nurse administrators should develop factors that motivate nurses to overcome the obstacles of patient education [27]. Nurse administrators assessed that the most important factor in forming motivation was to improve nurses' mentality and motivation [22]. When asked about educational barriers, nurses reported that managers should appreciate nurses' efforts in patient education [27] and provide feedback [27, 29].

*(2) Prioritizing Patient Education*. Patient education was not given enough attention by nurse administrators. However, both nurses and managers stated that everyone within an organization needs to actively participate in patient education and that patient education should be a priority [27]. Some nurses agreed that if managers did not value patient education, nurses should not be so concerned about it either [25].

*(3) Providing Support*. Nurse administrators reported the importance of helping new nurses with patient education [28]. According to nurse administrators, the most important factor in engaging nurses in patient education was utilizing nurses' suggestions for patient education [22]. Nurses' perceptions of managerial support for patient education include managerial consultation, valuing and supporting the nurses' advisory role [29], and interested and involved managers [24].

#### 4.3.2. Theme 2: Ensure the Necessary Resources for Patient Education

This theme describes nursing administrative actions to ensure all the necessary resources in patient education in three subthemes: creating facilities for patient education [22, 27, 28, 30], managing human resources [22, 25, 26, 28], and educating and training [26, 27]. In the next paragraphs, these subthemes will be described in detail: first, how nurse administrators saw themselves, second, highlighting the problems related to patient education, and third, what was seen as important for nurses.

*(1) Creating Facilities for Patient Education*. Nurse administrators saw their responsibility as creating facilities for nurses to provide patient education, such as providing space for education and access to the Internet [22, 28], and introducing standard forms for patient education [22]. Designing appropriate forms was seen as improving patient education [27]. Creating facilities for patient education also includes budgeting, where nurse administrators experience powerlessness in explaining deficits to their superiors [28]. Also, nurses reported that having designated facilities and rooms for patient education would enhance its effectiveness [27].

*(2) Managing Human Resources*. Human resource management was highlighted as a problem related to patient education by nurse administrators. They described situations of understaffing and tight work schedules that they had to manage [26–28]. Most of the nurse administrators still assessed that requesting nurses to provide patient education is realistic [25]. Establishing coordination in relationships and coordination of educators was assessed as an important task of the nurse administrators' role in patient education [22]. Nurses also reported factors relating to managing resources, and the lack of these was seen as a problem [27].

*(3) Educating and Training for Staff*. Educating and training new nurses was considered vital to improve their knowledge for effective patient education [27]. Nurse administrators reported that on-the-job staff training had been poorly realized [26]. Reflection was also an important part of education: one nurse administrator reported the importance of creating opportunities for nurses to reflect on their own care and patient education to support them grow as professionals [28].

#### 4.3.3. Theme 3: Enhance Patient Education Policies

The third main theme was enhancing patient education policies. Patient education policies were divided into international, national, organizational, and program levels [30]. These policies influence nursing administrative actions within an organization. The nursing administrative actions related to this main theme include three subthemes: monitoring and supervising patient education [23, 27–29], revising job descriptions [27, 28], and developing and implementing policies and procedures [22, 30]. In the next paragraphs, these subthemes will be described in detail; first, how nurse administrators supervise patient education, second, what nurse administrators mentioned should be revised, and third, what are the nursing administrative actions at different levels relating to developing and implementing policies and procedures.

*(1) Monitoring and Supervising Patient Education*. Nurse administrators supervise and monitor the patient education process [23, 27–29]. This also includes documentation to meet legal requirements and quality assurance, for which managers are responsible for supervising routine tasks and deciding on content [28]. Nurses reported that patient education requires constant supervision [27], although too much control was also perceived as limiting professional work [29].

*(2) Revising Job Descriptions*. Nurse administrators reported that their job descriptions need revision [27], e.g., by reducing the number of tasks [28], because they did not have sufficient time resources to fulfill their responsibilities [27, 28]. The nurse administrators also described situations where nurses' job descriptions need to be revised. Nurse administrators commented that if nurses had more time for education, the number of patient complaints about patient education would decrease [28].

*(3) Development and Implementation of Policies and Procedures*. Nursing administrative actions at different levels include developing policies for patient education programs and coordinating quality projects in patient education [30]. They also considered providing legal and professional information on patient education as an essential factor [22]. Furthermore, nursing administrative actions include implementing patient education policies, e.g., focusing on informed consent and promoting patient education through international organizations. At the national level, national laws, policies, and guidelines influence patient education. Centers of expertise and patient organizations influence patient education through quality projects. At the international level, the World Health Organization (WHO) promotes patient education through various projects [30].

## 5. Discussion

This integrative literature review examined the nursing administrative actions related to patient education, as there is a dearth of research in this area although it is a core nursing activity. The objective was reached, and the review produced information for both purposes: to inform healthcare organizations about the identified nursing administrative actions connected to patient education and to identify knowledge gaps for future research.

Different nursing administrative actions were identified from different perspectives, even though the number of analyzed studies was not very high (nine studies). Three main themes were formed: (1) strengthen the commitment to patient education, (2) ensure the necessary resources for patient education, and (3) enhance patient education policies. These themes highlight the actions corresponding to previous research on the standards developed for patient education [7] and nurse administrators' work content [13]. Additional research is needed on nursing administrative actions of patient education and to explore the connections and outcomes of these administrative actions to support high-quality and sustainable patient education.

Most of the studies focused specifically on the perspective of nurse administrators [22, 23, 25–29] working at the first and middle levels of their organizations; therefore, the identified actions do not necessarily represent all nursing administrative actions of patient education. First- and middle-level nurse administrators provided only a microview of detailed information on administrative actions related to patient education in the organizations. Moreover, the two qualitative studies included in this review showed that these nurse administrators did not always recognize the importance of their role in improving patient education [27, 28] which highlights the need to explore in more detail nurse administrators' experiences of their professional responsibilities concerning patient education. In previous research, nurse managers also find it difficult to express how to support nurses in undertaking fundamental care, possibly because it is overlooked in priorities [31]. The research area, therefore, still has gaps, and further research is needed in the future to synthesize these findings and explore reasons in depth. High-level administrators' actions on patient education need to be explored more, especially as there is a demand for nurses in high-level administration [32, 33]. Future research should take these different levels into account to provide a shared understanding of nursing administrative actions related to patient education. It would also be useful to analyze these different levels of administration actions separately to obtain more detailed practical information on patient education-related nursing administrative activities in organizations.

In the literature, the nursing administrative actions were not related to patients. For patients, the ethical and often legal basis for patient education is their right to be knowledgeable about their health and care [34], and therefore, for the administration of patient education, patient outcomes should be the main interest in nursing administrative actions and should not be disregarded in future research. In the future, research is needed to explore the connections between nursing administrative actions and patient outcomes. Also, the perspectives of patients about nursing administrative actions on patient education were lacking in the included studies. This may be due to the fact that administrative actions might not be directly visible to patients, although they may have useful views that can be relevant to nursing administration [35]. The importance of the patient's perspective is emphasized especially due to the growing interest in patient and public involvement [36] and administrators' efforts aimed at sustainable and transparent healthcare [37]. Including patients' views could potentially provide information on the outcomes of nursing administrative actions for patients.

Our aim to analyze the nursing administrative actions of patient education also deserves a critical comment. A challenge in the analysis was that the nursing administrative actions of patient education were not always clearly described, but the studies described problems relating to patient education. This was the assumption, and therefore, a broad selection of keywords was used. We tried to reach as wide a range of studies as possible but still did not reach very specific studies on nursing administrative actions related to patient education. Both nurses and nurse administrators describe similar organizational barriers that hinder patient education [22, 24–29]. A common barrier was lack of time [24, 25, 27–29] which has also led to missed nursing care in other studies as tasks with direct patient care have been prioritized over patient education [5, 38]. It is worth noting that nurse administrators experienced the same problems on which they should provide support for their nursing staff. Nurse administrators indicated that there was a lack of time for nurses to provide patient education due to tight work schedules [25, 28]. However, lack of time and constant changes also hinder administrators' work to support patient education [28]. These findings highlight the need for improvement in these areas. Nurse administrators should also be offered support in their work to enable them to act on patient education. The studies identified some actions by nurse administrators to address these issues, e.g., revising job descriptions, but there is still a lack of knowledge on how to solve these issues in more detail.

This integrative review explored comprehensively nursing administrative actions related to patient education. The number of studies on the topic was quite low, which may indicate that patient education has not been identified as an area of nursing administration. Despite the low number of studies, these different designs provide rich information about nursing administration related to patient education. In the future, it would be relevant to explore the actions identified in this review individually, targeting a specific activity, such as human resource management. It would also be beneficial to explore the connection between administration styles and patient education as it is known that there is a significant correlation between positive leadership styles and nurses' commitment to their work [16]. This could provide information on how to tackle the barriers to strengthening commitment to and ensuring the necessary resources for patient education.

### 5.1. Limitations

There are some limitations in this review, related to the sample/respondents, analysis of the result, and literature search. Respondents were first- and middle-level nurse administrators in their organizations, which may bias the generalizability of the results. Furthermore, the nursing administrative activities related to patient education were not always clearly described, which may have led to omissions in the analysis. However, this was controlled by monitoring the collection process and by frequent discussions within the research team. In addition, the quality of the included studies varied, and no report was excluded based on the data evaluation. In the literature search, the search terms “management” and “supervisor” generated a considerable number of studies of disease or patient management and supervising students, respectively. However, these were excluded at the latest in the full-text screening phase because they were out of the administrative scope of this review. In screening for the studies, the research team had constant discussions on differentiating the studies that focused directly on the nursing administrative action of patient education from those that reported administration actions in a limited section of the report.

## 6. Conclusion

This review provided new insight and a better understanding of nursing administration in patient education. Different nursing administrative actions related to patient education were identified relating to strengthening the commitment to patient education, ensuring that the necessary resources are available, and improving the policies for patient education. These actions highlight the important role of nurse administrators in patient education and should be made more visible. However, further research is needed to assess what are the outcomes of these nursing administrative actions on patient education. The results also revealed various obstacles related to these administrative actions, and these should be explored in the future.

## 7. Implications for Nursing Management

Based on the results of this review, nurse administrators tend to deprioritize patient education as their administrative action, or they do not have optimal conditions to act upon it. This review can raise awareness among nurse administrators on how to administer one of the central nursing actions and improve its quality. Nursing administrative actions vary, including strengthening the commitment to patient education, ensuring the necessary resources, and enhancing patient education policies. All these activities should be given high consideration when developing patient education in organizations. The implications for nursing management also include supporting future research in this area: the development of patient education and its management requires more scientific evidence.

## Figures and Tables

**Table 1:** Inclusion and exclusion criteria.

Inclusion criteria	Exclusion criteria
The main topic of the report is nursing administrative actions related to educational actions	The main topic of the report is nurse administrator-developed or researched patient education (but not nursing administrative activities)
Educational actions are connected to health	
Individual or group health education or promotion	Health promotion at the population/community level
An international scientific peer-reviewed report	Not scientific peer-reviewed report, e.g., conference abstracts, theses/dissertations, editorials, and letters
Empirical research report	Discussion/theoretical papers and literature review
The full text is available	
English language	

**Table 2:** An example of the search strategy (PubMed).

(“Health Education” [Mesh] OR “Health Promotion” [Mesh] OR “Consumer Health Information” [Mesh] OR “Patient Education as Topic” [Mesh] OR “Counseling” [Mesh] OR “health education” [tw] OR “patient education^*∗*^” [tw] OR “patient-educat^*∗*^” [tw] OR counseling [tw] OR counseling [tw] OR “health information^*∗*^” [tw] OR “health promoti^*∗*^” [tw] OR “patient guidance” [tw] OR “patient information” [tw] OR “patient teach^*∗*^” [tw] OR “treatment information” [tw] OR “health advice” [tw]) AND (“Nurse Administrators” [Mesh] OR “Nursing Administration Research” [Mesh] OR “Nursing, Supervisory” [Mesh] OR “nurse leader^*∗*^” [tw] OR “nursing leader^*∗*^” [tw] OR “nurse manager^*∗*^” [tw] OR “nurse management^*∗*^” [tw] OR “nursing management^*∗*^” [tw] OR “nurse executive^*∗*^” [tw] OR “nurse administrator^*∗*^” [tw] OR “nursing administrator^*∗*^” [tw] OR “head nurse^*∗*^” [tw] OR “nurse supervision” [tw] OR “nursing supervision” [tw])

**Table 3:** A summary of the selected studies (*n* = 9).

Author(s), year of publication, and location	Purpose of the study	Sample, sample size, and sampling method	Study design and data collection	Context	Data analysis	Quality scores (JBI 2020, critical appraisal tools)
Fereidouni et al. 2019, ira [27]	“To document the perspectives and recommendations of nurses with regard to patient education” p.2	Head nurses, *n* = 8, clinical nurses, *n* = 16, and purposive sampling	A qualitative exploratory design semistructured in-depth interviews focus group observation	Medical, surgical, emergency, and pediatric departments	Conventional content analysis (Graneheim and Lundman 2004)	7/10

Ghorbani et al. 2019, Iran [22]	“To determine the mechanisms for attracting nurses” engagement in patient education from the viewpoint of nursing managers” p.164	Nursing managers (ward head, supervisor), *n* = 91, convenience sampling	A cross-sectional descriptive-analytic study A two-part questionnaire (developed for this study) (40 questions)	Educational hospitals	Descriptive statistics methods	7/8

Bergh et al. 2015, Sweden [28]	“To explore the conditions for nurses' daily patient education work by focusing on managers' way of speaking about the patient education provided by nurses in hospital care” p.192	Managers, *n* = 10	A qualitative exploratory design (i) A social constructionist perspective 3 focus group interviews	Not specified, patient education in hospital care	Critical discourse analysis	9/10

Seyedin et al. 2015, Iran [23]	“To investigate the dimensions of patient education process including need assessment, planning, implementation, and evaluation” p.2	Head nurses, *n* = 187, convenience sampling	A descriptive cross-sectional study A questionnaire (developed for this study) (i) 1 part: demographics, (ii) 2 part (31 items) a five-point Likert scale (1 = never and 5 = always)	Teaching hospitals	Descriptive statistics methods	4/8

Bergh et al. 2012, Sweden [24]	“To describe nurses' perceptions of conditions for patient education, focusing on organizational, environmental and professional cooperation aspects” p.759	Nurses, *n* = 701, stratified random sample	A cross-sectional survey A questionnaire (developed for this study) (60 items) with fixed response categories (1–5) dichotomous response options (yes or no) open-ended answers	Primary care, municipal care, and hospital care	Quantitative data: statistics methods Qualitative data: a content analysis	8/8

Vafaee-Najar et al. 2012, Iran [25]	“Studying what patient education services were offered and which organizational factors in the hospitals affected the provision of these services” p.231	Patients, *n* = 441, physicians, *n* = 200, nurses, *n* = 185, supervisors, and managers, *n* = 70, stratified and simple sampling	A descriptive cross-sectional study of four self-administered questionnaires Three answer choices (agree, disagree, and neutral) open-ended questions and closed questions interviews	Teaching hospitals	Quantitative data: statistics methods Qualitative data: a content analysis	2/8

Hätönen et al. 2010, Finland [26]	“To describe patient education practices in adult acute psychiatric hospitals” p.334	Head nurses from adult acute psychiatric wards, *n* = 55	A descriptive questionnaire survey A questionnaire (developed for this study) 7-point scale dichotomous response options (1 = yes or 2 = no) open-ended question	Adult acute psychiatric hospitals	Quantitative data: statistics methods Qualitative data: a content analysis	6/8

Valanis et al. 2003, U. S [29]	“Describes observed variations in telephone advice nursing services and the organizational and process factors the nurses identified as supporting or hindering their work” p.216	Taped (1 to 2 hours) calls: call centers, *n* = 77 medical offices, *n* = 98, nurses and physicians, (*n* = not reported), managers, (*n* = not reported), a convenience sample	A qualitative design Two instruments (developed for this study) (i) The call description form (ii) The interpersonal communication (iii) Style inventory manager-completed checklists focus groups (nurses and physicians)	Telephone advice nursing services	Qualitative analysis	5/10

Albada et al. 2001, Netherlands [30]	“Describes the organization of patient education in hospitals and the conditions that influence this in the Netherlands, Flanders and England” p.4	Patient education officers, an executive of a health insurance company, Ombudsman, a committee member of a professional organization, *n* = 5, document analysis, *n* = 95, documents and websites, *n* = 24	Qualitative design document analysis of five interviews	Not specified, patient education in hospitals	Text coding analysis document analysis	5/10

**Table 4:** Main themes, subthemes, codes, and original phrases describing nursing administrative actions related to patient education.

Subtheme	Code	Original phrase	Study
*Main theme 1: Strengthen the commitment to patient education*			
Enhancing motivation for patient education	Developing factors that motivate nurses	“To overcome the obstacles of patient education, total commitment is necessary. Nurses must be fully willing to devote the necessary time and energy to patient education and believe in it. Thus, administrators should strengthen institutional commitment by developing motivational factors and facilitating change by every impetus to achieve this milestone.” p.4	Fereidouni et al. 2019
	Motivating nurses	“Regarding motivation, the most important factor from the viewpoint of head nurses was forming motivation to follow the policy and teaching methods to the patient.” p.166	Ghorbani et al. 2019
	Improving nurses' motivation	“Regarding motivation, the most important factor from the viewpoint of supervisors was Improving the mentality and motivation of nurses.” p.166	Ghorbani et al. 2019
	Appreciating nurses' efforts in patient education	“In another case, a participant stated that ignoring the nurses who enroll eagerly in patient education is not reasonable. Managers should appreciate their efforts, even with a smile or other acknowledgment.” p.4	Fereidouni et al. 2019
	Improving patient education motivation and performance	“Nurses indicated they did not receive feedback on advice appropriateness and that feedback on caller outcomes would be more helpful than the monitored service indicators.” p.220	Valanis et al. 2003

Prioritizing patient education	Considering patient education an important factor	“12.2% of the nurses agreed with the viewpoint that if the managers did not consider patient education to be an important factor they should also not be so concerned about it.” p.234	Vafaee-Najar et al. 2012
	Considering patient education a priority	“The participants stated that all stakeholders should consider patient education a priority and take measures toward improving education. One participant mentioned: “A lasting change requires a general determination. All people in the organization from the top of pyramid to its base should actively participate”.” p.4	Fereidouni et al. 2019
	Focusing on patient education	“The observations also revealed that these supervisors did not pay attention to patient education in their rounds and did not consider this domain to be a priority. The following field note showed that practical involvement of the supervisors was not sufficient.” p.4	Fereidouni et al. 2019
	Dedicating special staff to patient education	“71.4% believed that due to the nurses' workload in each division, patient training would only be possible if special staff were dedicated to it.” p.234	Vafaee-Najar et al. 2012
	Paying more attention to helping young nurses	“It's important that managers have an opportunity to help young nurses instead of being overwhelmed by administrative tasks—we're the ones who should help them. Patient teaching takes time, thus it's a question of where we're going to find the time.” p.194	Bergh et al. 2015

Supporting patient education	Leveraging nurses suggestions for patient education	“Using nurses' constructive and effective suggestions for patient education, from the viewpoint of supervisors” p.166	Ghorbani et al. 2019
	Supporting the nursing profession	“Nursing personnel complained about their authority and decision making in hospitals. “Favoritism is corrosive. Desecration, discrimination, lack of authority, and professionalism are disappointing in our hospital. First of all, we should strengthen our profession and define our territory by choosing a qualified matron, supervisors, and nursing personnel”.” p.4	Fereidouni et al. 2019
	Getting interested and involved in supporting patient education	“A total of 214 nurses responded to the supplementary open-ended item regarding perceptions of managerial support in nurses' PE. Five categories emerged: offer of professional competence development, allocated time, available room for teaching, available working tools and interested and involved managers.” p.761	Bergh et al. 2012
	Providing consulting options for nurses executing patient education	“One nurse observed that “I am not trained to deal with suicidal patients and yet, after hours, I am the only one available in the system to deal with this member.” Although she did have access to the on-call physician or could refer the patient to outside services, she still felt unsupported. In the medical offices, nurses could consult with an on-site physician, pharmacist, or supervisor.” p.220	Valanis et al. 2003
	Valuing and supporting the nursing advice role	“Nurses expressed varying perceptions of the extent to which the healthcare system valued and supported the nursing advice role. One medical office nurse stated: “I wonder how much advice nurses are actually valued. If there is a staff shortage in the clinic, we are always the first ones pulled”.” p.221	Valanis et al. 2003

*Main theme 2: Ensure the necessary resources for patient education*			
Creating facilities for patient education	Ensuring adequate working conditions	“Managers regarded themselves as responsible for creating the necessary conditions for nurses to conduct their daily work.” p.194	Bergh et al. 2015
	Creating room and facilities	“The importance of barriers to patients' education, the most important factor from the perspective of head nurses was the creation of facilities and classes for patient education (video-internet-lag).” p.166	Ghorbani et al. 2019
	Facilitating patient education work	“There was a desire for external parties to study the managers' work situation to help them facilitate nurses' patient education work: “We're running around and an outsider would surely ask: Why are you doing that?”.” p.196	Bergh et al. 2015
	Providing room to conduct patient education	“We need a quiet room in our wards for patient education to enhance its effectiveness. Concentration in a noisy environment is impossible for patients.” p.5	Fereidouni et al. 2019
	Providing facilities to conduct patient education	“We need some facilities (such as video, TV, video software, and so on) to show various educational films on patient diets and medications while patients are in beds in their own rooms.” p.5	Fereidouni et al. 2019
	Budgeting	“The managers were powerless as professionals in relation to their superiors when budgeting was discussed: “‘We're virtually up against the wall when we have to explain our deficits”.” p.196	Bergh et al. 2015
	Implementing standardized patient education forms	“From the viewpoint of head nurses, included introducing standard forms of education to the patient provided by health Ministry (3.88 ± 0.97), while it was being aware of the actual and potential capabilities of nurses (3.91 ± 0.79).” p. 166	Ghorbani et al. 2019
	Designing forms for patient education	“The participants stated that having a specific form for patient education was necessary. They mostly mentioned that the current forms that were used in the hospital were obligatory for patients and quite time-consuming to complete. Thus, they recommended designing appropriate forms to improve patient education.” p.4	Fereidouni et al. 2019
	Developing patient education leaflets	“In Dutch hospitals on the program level of patient education, there are specialized nurses with important roles in patient education for several patient groups. Dutch hospitals have a large number of patient leaflets on treatments that are developed within the hospital.” p.6	Albada et al. 2001
	Managing patient education materials	“Most Flemish hospitals have little organization of patient education on the organizational level. The editing of patient information leaflet is an important activity on the organizational level. This task is generally among the responsibilities of a general communications officer.” p.7	Albada et al. 2001
	Developing patient education leaflets	“The hospital developed patient education leaflets for diabetes and cataract patients. Policy on when to give face-to-face information and leaflets is present for education with diabetes patients.” p.7	Albada et al. 2001

Managing human resources	Managing resources and environmental conditions	“Altogether 50 respondents described problems related to patient education on their wards. First, patients' poor condition in terms of lack of insight and poor motivation was perceived most frequently (*n* = 33) to hinder the patient education. Second, a lack of staff resources (*n* = 25) meant that there was not sufficient staff on the wards and they were not motivated or competent to carry out patient education. Third, the discrepancy in the procedures (*n* = 17) concerned the unplanned and short treatment periods and lack of patient education instructions. Fourth, poor operational conditions (*n* = 9) were described.” p.337	Hätönen et al. 2010
	Coping with tight nursing schedules	“A typical description of the connection between available resources and nurses' patient education work is provided in the following quotation: “It mustn't cost anything. We struggle with the tight nursing turnarounds where everything has to be done incredibly quickly. We might have to discharge 6-7 patients every day and it's almost like a conveyor belt, but what kind of information do they get? There used to be a discharge meeting, at which the patient could ask the physician and the nurse questions but that rarely happens today. When the patient asks questions in connection with discharge, the nurse often says—yes but you saw the physician” [in the nurse's opinion the patient should have asked the physician at the discharge meeting].” p.196	Bergh et al. 2015
	Requesting nurses to provide patient education	“17.6% of the managers also believed that “Due to the high volume of responsibilities expected from nurses, requesting them to provide a patient education service is unrealistic”.” p.234	Vafaee-Najar et al. 2012
	Managing shifts	“Considering the position of participatory management, the most important factor from the perspective of head nurses was the coordination in relationship and coordination of trainers in different shifts.” p.166	Ghorbani et al. 2019
	Managing available resources	“Other obstacles to the development of patient education were that nurses often changed workplaces and that available healthcare resources were reallocated and used for training.” p.196	Bergh et al. 2015
	Coping with a shortage of nurses	“I think that patient education falls through the cracks due to the high workload in the hospital. The nursing shortage is really a crisis. When I have seven patients in each shift, there is no chance left for patient education.” p.5	Fereidouni et al. 2019

Educating and training	Educating and training new nurses	“Participants stated that newly graduated students were novices and that they started working in hospitals without experience. Thus, these new nurses needed to understand the importance of patient education and be thoroughly trained in this regard. One participant stated, “Frequent in-service education is recommended for nurses to improve their knowledge to educate patients efficiently”.” p.5	Fereidouni et al. 2019
	Improving training for staff	“Less than half of the (45%) respondents reported that the nurses had sufficient professional education to deliver patient education interventions, whereas sufficient on-the-job training for staff had been poorly realized.” p 337	Hätönen et al. 2010
	Providing opportunities for professional growth	“Previously, nurse educators participated in students' caring activities. Caring conversations created conditions for nurses to reflect over their own care and patient education, thus helping them grow in their profession. The managers stated that it was necessary for nurses to have time for reflection: “to advance from novice to expert” (FG, 2), something that was missing today.” p.196	Bergh et al. 2015
	Offering professional competence development in patient education	“Primary care managers offered professional competence development in PE significantly more frequently than in MC/HC.” p. 761	Bergh et al. 2012

*Main theme 3: Enhance patient education policies*			
Monitoring and supervising patient education	Giving positive and negative reinforcement	“A head nurse said, “Managers should use carrots and sticks at the same times. Frequent punishment will not work”.” p.4	Fereidouni et al. 2019
	Monitoring nursing tasks	“After repeated prompts to provide a more detailed description of the prerequisites for patient education, the managers quickly moved to general descriptions of nursing tasks that have to be monitored, such as documentation and liaison.” p.197	Bergh et al. 2015
	Supervising the process of patient education	“On the other hand, they are the line managers in hospitals who directly supervise the processes; therefore, they know and can judge the process of patient education in their wards.” p.2	Seyedin et al. 2015
	Ensuring quality	“The managers have control over routine matters as well as the power to decide the content, which implies quality assurance.” p.197	Bergh et al. 2015
	Documenting	“The juridical discourse contained several controlling factors that restricted the nurses' time available for patient education. By referring to the public regulations in health care, the managers expressed that nurses must exercise control and be controlled. The regulations require documentation. “I'm not exactly terrified but “I have to document everything to cover my back” is frequently heard”.” p.197	Bergh et al. 2015
	Monitoring the implementation of patient education	“The participants acknowledged that including the implementation of patient education in annual personnel evaluations would be effective. Another participant also mentioned that “annual supervision at a specific time is not effective. Supervision should be intrusive and frequent”.” p.4	Fereidouni et al. 2019
	Paying attention to how patient education is monitored	“Nurses reported aspects of the practice environment that limited their professional practice, impaired their optimal functioning, and contributed to low morale. These included the extent to which they were required to adhere to protocols, supervisor emphasis on time targets (eg, call time, talk time, and documentation time), and monitoring to ascertain that required questions were asked of all callers.” p.221	Valanis et al. 2003
	Managing operations	“Hence, they expressed a desire to have power to manage operations.” p.196	Bergh et al. 2015

Revising job description	Reducing the number of tasks of managers	“Constant changes constituted an obstacle to the development of the patient education provided by nurses, and all professional categories had tasks that were beyond their professional competence. The managers had no time. Only the most important administrative tasks were performed, and the rest were ignored: “It depends on how you sell it and your own opinions. I really resist any change. we're fire fighters, we only have time to man the ward. We can't reorganise things and think afresh. We have done that; now we have to reduce the number of tasks”.” p.196	Bergh et al. 2015
	Updating the description of head nurses' tasks and responsibilities	“All of the head nurses mentioned that their job description needed to be revised by their hospitals based on new accreditation criteria. They stated that they were currently busy with the new accreditation criteria and standards, which led to their unintentional neglect of nursing care obligations such as patient education.” p.4-5	Fereidouni et al. 2019
	Clarifying nurses' job descriptions	“Managers commented that patient criticism about not receiving or understanding information would decrease if nurses devoted more time to patient teaching: “They do a whole lot of the physicians” tasks these days. Many physicians don't prescribe tests—the nurse has to do it in our computer system and decides which tests should be performed. A lot of time is spent changing prescriptions for medications that are completely off the wall. if the nurse didn't have to do these medical tasks she'd have more time for patient education”.” p.197	Bergh et al. 2015

Developing and implementing policies and procedures	Providing legal and professional information on patient education	“Informing nursing staff on legal and professional issues regarding avoiding education to patients from the viewpoint of supervisors.” p.166	Ghorbani et al. 2019
	Developing education policies	“Policy on face-to-face education is present in Dutch hospitals for some patient groups and is developed in multidisciplinary projects supported by a patient communications officer.” p.6	Albada et al. 2001
	Coordinating projects	“Some hospitals have quality projects in patient education. The communications officer is a central person within this network. The coordination of the network, editing patient leaflets, and coordinating quality projects in patient education are among the responsibilities of the communications officer. Until recently these were the tasks of a patient education coordinator, but this function no longer exists.” p.7	Albada et al. 2001
	Implementing ombuds services in hospitals	“In 2002, the Law on Patient's Rights stipulated that every hospital should have an ombuds service [17]. The ombuds services have now been implemented in hospitals. The law also set out guidelines for patient education but these guidelines do not greatly influence practice.” p.7	Albada et al. 2001
	Implementing programs	“Medical Treatment Contract Act (WGBO) has made it the duty of healthcare workers to provide their patients with information [11–13]. This law greatly stimulated the policy and practice of patient education in hospitals and heralded the start of a broad implementation program that focused on informed consent.” p.6	Albada et al. 2001
	Lobbying	“This patient organization has a stimulating influence on the organization of patient education through lobbying and quality projects in health care.” p.6	Albada et al. 2001
	Promoting patient education through international organizations	“The World Health Organization (WHO) promotes patient education through the Health Promoting Hospitals project.” “The European Association for Communication in Healthcare (EACH) aims to stimulate research and education on communication in healthcare.” “The European Association for the Study of Diabetes (DESG) provides advice and best practices on patient education in diabetes.” p.8	Albada et al. 2001

## Data Availability

The data supporting this integrative review are from previously reported studies and datasets, which have been cited.
